# Dual-Bayes Localization Filter Extension for Safeguarding in the Case of Uncertain Direction Signals

**DOI:** 10.3390/s18103539

**Published:** 2018-10-19

**Authors:** Alexander Brunker, Thomas Wohlgemuth, Michael Frey, Frank Gauterin

**Affiliations:** 1 Department of Parking Systems, Daimler AG, 71063 Sindelfingen, Germany; hh1714@partner.kit.edu (A.B.); thomas.wohlgemuth@daimler.com (T.W.); 2 Institute of Vehicle System Technology, Karlsruhe Institute of Technology, 76131 Karlsruhe, Germany; frank.gauterin@kit.edu

**Keywords:** Bayes filter, driving state estimation, direction detection, pattern recognition, equilibrium of forces, slope

## Abstract

In order to run a localization filter for parking systems in real time, the directional information must be directly available when a distance measurement of the wheel speed sensor is detected. When the vehicle is launching, the wheel speed sensors may already detect distance measurement in the form of Delta-Wheel-Pulse-Counts (DWPCs) without having defined a rolling direction. This phenomenon is particularly problematic during parking maneuvers, where many small correction strokes are made. If a localization filter is used for positioning, the restrained DWPCs cannot process in real time. Without directional information in the form of a rolling direction signal, the filter has to ignore the DWPCs or artificially stop until a rolling direction signal is present. For this reason, methods for earlier estimation of the rolling direction based on the pattern of the incoming DWPCs and based on the force equilibrium have been presented. Since the new methods still have their weaknesses and a wrong estimation of the rolling direction can occur, an extension of a so-called Dual-Localization filter approach is presented. The Dual-Localization filter uses two localization filters and an intelligent initialization logic that ensures that both filters move in opposite directions at launching. The primary localization filter uses the estimated and the secondary one the opposite direction. As soon as a valid rolling direction signal is present, an initialization logic is used to decide which localization filter has previously moved in the true direction. The localization filter that has moved in the wrong direction is initialized with the states and covariances of the other localization filter. This extension allows for a fast and real-time capability to be achieved, and the accumulated velocity error can be dramatically reduced.

## 1. Introduction

In order to achieve a robust and precise positioning of intelligent vehicle systems, localization filters which connect different vehicle and sensor models were developed by multi-sensor data fusion. In the literature, different sensor models of the wheel speed sensors [1,2,3], the steering wheel sensors [2], and the yaw rate sensors [3,4] are used to model the vehicle’s motion. For parking systems, the sensor models of the wheel speed sensors are particularly important, as parking faces the challenges of rolling direction changes, which have to be detected through the wheel speed sensor itself. Bayesian filters are used to fuse the different sensor information together. These filters use the information in real time and react extremely sensitively to systematic and unsystematic errors. The structure of these filters by weighted average estimation means error reduction by fusion, but also means that they react sluggishly. Problems arise when the filter uses an incorrect estimated rolling direction because it is very expensive to perform the faulty calculation retroactively. The filter extension introduced in this paper is intended to help in inaccurate rolling direction estimation. The importance of the use of filter extensions and their continuous further development would be clearly demonstrated by their application in modern and sophisticated filter techniques, using, for example, out-of-sequence measurement filters [5], interacting parallel filters [6], particle learning filters [7], or group importance sampling filters [8].

**Figure 1:**
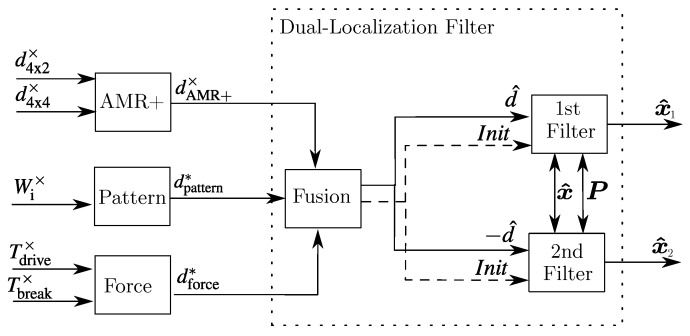
Rolling direction estimation and Dual-Localization filter concept.

This paper introduces a Dual-Localization filter method that allows two filters to run in the opposite rolling direction when they are put into operation to run in real time. When launching, it happens that the wheel speed sensors already detect an information about the velocity in the form of Delta-Wheel-Pulse-Counts (DWPCs) without having defined a direction. The localization filter cannot process these DWPCs with an undefined rolling direction signal in real time and would ignore or artificially stop these measurements until a valid rolling direction signal is present. For this reason, in the patent application [9], a procedure for determining a rolling direction change based on the pattern of the incoming DWPCs and in the patent application [10], a method for detecting the rolling direction by a force equilibrium in the longitudinal direction of the vehicle is presented. Since the new methods still have weaknesses and a wrong estimation of the direction can occur, an extension of the localization filter that ensures that a continuous calculation of the position can take place by a localization filter has been developed, even if the considered rolling direction has turned out to be wrong. This idea of a so-called Dual-Localization filter has already been presented in patent application [11] and in this paper a possible application and implementation is shown. The Dual-Localization filter uses two localization filters and an intelligent initialization logic that ensures that both filters always move in the opposite direction when launching. The first so-called primary localization filter uses the estimated direction and the secondary one uses the opposite direction. As soon as a valid rolling direction signal is present, an initialization logic is used to decide which localization filter has previously moved in the true direction. The localization filter that has moved in the wrong direction is initialized with the states and covariances of the other localization filter. The basic structure of the different rolling direction estimations and the Dual-Localization filter is visualized in Figure 1.

The division of this work is organized as follows: In Section 2, the localization filter is briefly introduced. Section 3 shows the different possibilities to detect the rolling direction. The evaluation of the rolling directions and the initialization logic is presented in Section 4. Finally, experimental results are shown in Section 5, where the performance is demonstrated in real test scenarios from parking and real test scenarios on the reference gradient hill. For the following formulations, a time-discrete description with the index k is selected. For all wheels of the vehicle, the index i∈{FL,FR,RL,RR} is chosen. Sensor measurements or quantities derived from the sensor model are illustrated with ${}^{\times }$ and empirically corrected quantities are illustrated with ${}^{*}$.

## 2. Localization Filter

For vehicle localization, a filter merges different dead-reckoning models together. To enable a robust and precise estimation of the vehicle position, the information from different sensor measurements can be combined to obtain an exact estimate. The filter algorithms work as a kind of weighted average estimator and calculate the states and covariances. The covariances are the estimated errors of the states resulting from the possible errors of the measurements and inputs. The filters use these as a kind of memory, so to speak, and are thus in a position to determine very precise states even in the event of measurement errors. The filter delivers a trajectory consisting of the position $p_{k}={\left[x_{k}\;y_{k}\right]}^{T}$ and orientation $\theta_{k}$ in relation to a fixed starting point. In addition, the velocity angle $\beta_{k}$, the velocity $v_{k}$, and the yaw rate $\omega_{k}$ of the vehicle are delivered.

(1) $$\begin{array}{c} x_{k}={\left[\begin{array}{cccccc} x_{k} & y_{k} & \theta_{k} & \beta_{k} & v_{k} & \omega_{k} \end{array}\right]}^{T}. \end{array}$$

The measurements used in the filter $z_{k}$ include the individual wheel angles vector $\delta_{k}$, the individual wheel velocities vector ${\tilde{v}}_{k}^{\times }\left(d_{k}^{\times }\right)$, which is dependent on the rolling direction, and the yaw rate $\omega_{k}^{\times }$ measured by the yaw rate sensor.

(2) $$\begin{array}{c} z_{k}={\left[\begin{array}{ccc} \delta_{k} & {\tilde{v}}_{k}^{\times }\left(d_{k}^{\times }\right) & \omega_{k}^{\times } \end{array}\right]}^{T}. \end{array}$$

The localization filter then solves the problem of fusing different measurements with different models by using the Bayesian filter equations. There are several forms of Bayesian filter to choose from. The Kalman filter is certainly the best known. It uses two phases in which a system model performs a prediction of the state and in which the states are corrected by the measurement model in the innovation phase. Another form is the Information filter, which uses an inverse form of equation in the innovation phase and is advantageous if the system has more measurements than states. Both filters can be applied to non-linear samples using the extended method which uses the 1st Taylor series linearization or the unscented method which uses a linearization up to the 3rd Taylor series. In this work, an extended information filter (EIF) is used, which is well suited for this system due to its simplicity and fast calculation. The EIF calculating a new state *x* using the system model f and the measurement model h:

(3) $$\begin{array}{cc} {\hat{x}}_{k} & =f({\hat{x}}_{k-1}),\\ {\hat{z}}_{k} & =h\left({\hat{x}}_{k}\right). \end{array}$$

However, the filter itself is not part of this work and is therefore assumed as given. A more detailed description and application of the localization filter is discussed in the article [12].

## 3. Detection of Rolling Direction

**Figure 2:**
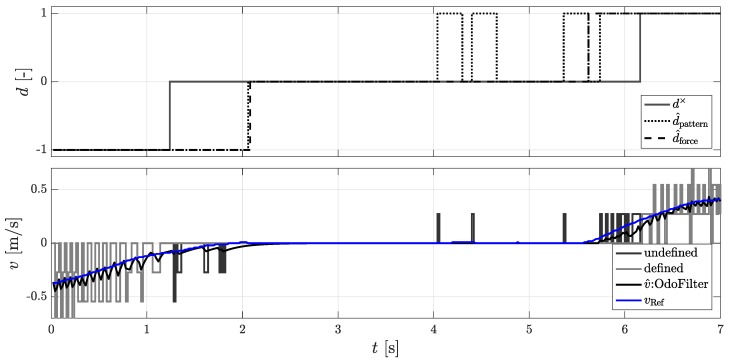
(**Top**): The rolling directions of the Delta-Wheel-Pulse-Counts (DWPCs) signal $d^{\times }$, the pattern recognition method ${\hat{d}}_{\mathrm{pattern}}$, and the force equilibrium method ${\hat{d}}_{\mathrm{force}}$; (**Bottom**): Undefined and defined DWPCs and velocity of the OdoFilter using the new rolling directions methods.

In Figure 2, the time line of the different rolling direction signals is represented. Here, the test vehicle was braked from a reverse drive to a standstill and accelerated forwards after a short time. As can be seen, the two methods of pattern recognition ${\hat{d}}_{\mathrm{pattern}}$ and force equilibrium ${\hat{d}}_{\mathrm{force}}$ allow the actual rolling direction to be recognized more quickly than with the DWPC signal $d^{\times }$.

In the following, the algorithms with their strengths and weaknesses will be described. A test vehicle was equipped with a Dual-DGNSS-IMU reference system consisting of two Differential-Global Navigation Satellite Systems (DGNSS) with RTK-differential data from ground-based reference stations (communication via LTE) and an Inertial Measurement Unit (IMU), which provides an accuracy of the measured velocity of 0.04 m/s.

### 3.1. Roll Direction Detection via AMR+ Sensors

Wheel speed sensors are divided into active and passive sensors, but currently it is almost always active wheel speed sensors that are installed for automotive applications. These sensors, with the anisotropic magnetoresistive (AMR) effect, measure the orientations of a magnetic field of a pole wheel. In the past, inductive passive sensors were also used to measure the magnetic field change over time. Active wheel speed sensors have the advantage of measuring low velocities, and every magnetic field change is detected [13].

### 3.2. Velocity Measurement

With the AMR sensors, the DWPC $w_{i,k}^{\times }$ can be determined for each wheel. The sensor counts the counter value $En_{i,k}^{\times }$ continuously up to the maximum counter value $En_{\max}=$ 255 and then starts again from 1. To calculate the DWPC, the modulo operation (the modulo operation mod(x,y) will give the rest after dividing x by y. It is assumed that, at a sampling interval of Δt= 0.02 s, the counter value is not more than $En_{\max}=$ 254 (from 0–254 = 255 steps), which would mean 255 m/s = 918 km/h at a rolling circumference of c= 2 m and a resolution of $w_{\max}=$ 100) is used:

(4) $$\begin{array}{cc} w_{i,k}^{\times } & =\mathrm{mod}\left[\left(En_{i,k}^{\times }-En_{i,k-1}^{\times }\right),En_{\max}\right]. \end{array}$$

The maximum number of DWPCs per revolution results from the number of magnet pole pairs $n_{\mathrm{Pol}}$ or the resolution of the wheel velocity sensor $A_{\mathrm{WHL}}$ to

(5) $$\begin{array}{c} w_{\max}=2\cdot n_{\mathrm{Pol}}=\frac{1}{A_{\mathrm{WHL}}}. \end{array}$$

The traveled path $\Delta s_{i,k}$ results from the rolled angle $\Delta \phi_{i,k}$ and the radius $r_{i}$ of the wheel:

(6) $$\Delta s_{i,k}=\Delta \phi_{i,k}\cdot r_{i}=2\pi \cdot w_{i,k}^{\times }\cdot A_{\mathrm{WHL}}\cdot \frac{c_{i}}{2\pi }=\frac{w_{i,k}^{\times }\cdot c_{i}}{w_{\max}}.$$

Using the direction of rotation $d_{i,k}^{\times }$ and the constant sampling time $\Delta t=1/f_{S}$, the wheel circumferential velocity ${\tilde{v}}_{i,k}^{\times }$ can be calculated:

(7) $$\begin{array}{c} {\tilde{v}}_{i,k}^{\times }=\frac{ds}{dt}=\frac{\Delta s_{i,k}}{\Delta t}\cdot v_{i,k}=\frac{w_{i,k}^{\times }\cdot c_{i}\cdot d_{i,k}^{\times }}{w_{\max}\cdot \Delta t}. \end{array}$$

Since the AMR sensors detect every reversal of the direction of rotation of the magnet poles, only the quantization error due to the resolution is decisive for the velocity measurement, which is taken into account when tuning the localization filter.

#### Rotational Direction Measurement

Modified active wheel speed sensors can also detect the rolling direction. The direction of rotation of these AMR+ sensors can be detected by a signal shift with two slightly twisted pole rings. For vehicles with only one driven axle, only the non-driven axle is equipped with AMR+ sensors for cost reasons. For vehicles with two driven axles, all four wheels are normally equipped with AMR+ sensors. The direction of rotation can be positive, negative, or undefined and is defined as follows:

(8) $$\begin{array}{c} d_{i,k}^{\times }\in \left\{-1,0,1\right\}. \end{array}$$

AMR+ sensors can detect up to three magnetic field changes when the vehicle is started up before it can provide a valid direction of rotation. As shown in Figure 2, there is only one valid rolling direction signal after the DWPCs have been measured (4 to 6.1 s). During measurements, a value of up to four DWPCs could be measured without the presence of a driving direction signal. An inaccuracy or time delay of the speed signals cannot be taken into account when tuning the localization filter. Therefore, the following methods were developed, which can determine a rolling direction with the first DWPC.

### 3.3. Roll Direction Detection by Pattern Recognition

**Figure 3:**
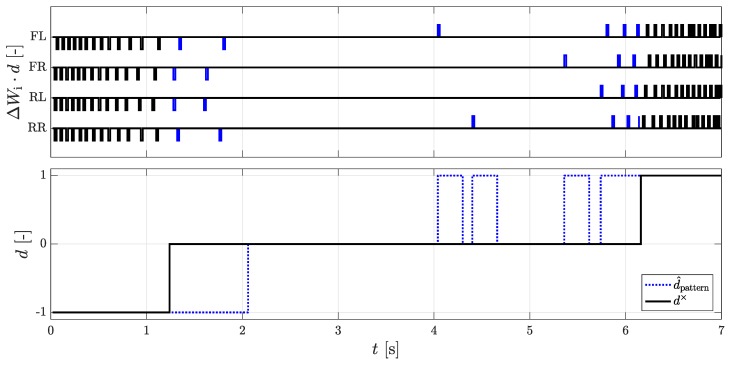
(**Top**): Undefined (blue) and defined (black) DWPCs using the pattern recognition method for detection of rolling directions; (**Bottom**): The rolling directions of the DWPC signal $d^{\times }$ and the pattern recognition method ${\hat{d}}_{\mathrm{pattern}}$.

For an early recognition of a change of the rolling direction, a procedure that investigates the chronological sequence of the DWPC of the individual wheels in pairs was developed. A detailed description of the procedure with an example can be found in the patent application [9]. The principle is similar to the direction recognition of the AMR+ sensors, where two encoder rings are used to detect a phase shift of the signals. In Figure 3, one recognizes that the DWPC for the time range t=1–2 s alternate with the following scheme [..., RL, FR, RR, FL]. The schema [FL, RR, FR, RL, ...] is then reflected, and the vehicle has changed its rolling direction. The first four DWPCs of the front left and the rear right wheel and the three DWPCs of the front right and rear left wheel have thereby no directional affiliation by the signal of the AMR+ sensors. It can also be seen that the direction of the driving is maintained for 250 ms in order to prevent fast direction changes.

The procedure is implemented by an algorithm which stores and evaluates the sequence of the DWPCs. For this purpose, six wheel pairs are formed, and a feature vector $F_{\mathrm{pattern},j,k}$ is determined. The system then checks, for each pair of wheels, whether the feature vector has been mirrored. If a reflection is detected for more than two wheel pairs, a change in the rolling direction is assumed. The chronological sequence results from a random rotation of the encoder rings of the wheels against each other. In the unlikely event that all DWPCs occur simultaneously because the encoder rings are perfectly aligned, the algorithm cannot detect any change in the rolling direction. With a change of the wheel angle by steering at standstill, it can also come to the detection of DWPCs. In this case only the wheel pair of the rear axle should be used. The rolling circumference difference between the wheels has a negligible influence on the sequence. The procedure can only detect a change in rolling direction, and there is no indication of the absolute rolling direction. The algorithm has its weakness when the roll circumference is very different or when it is steered at a standstill and the order changes.

### 3.4. Rolling Direction Detection by Force Equilibrium

By force equilibrium in the longitudinal direction of the vehicle, a rolling direction and a safe standstill can be determined. A detailed description of the procedure including algorithm with an example can be found under the patent application [10]. In the procedure described above, a velocity is derived from the force equilibrium:

(9) $$\sum_{\mathrm{veh}}F_{x,\mathrm{veh}}\left(v_{x}\right)=F_{\mathrm{drive}}-F_{\mathrm{fric}}\left(v_{x}\right)=m\cdot {\dot{v}}_{x}.$$

The sign of the velocity determines the rolling direction. If the velocity is 0 and the braking force is high enough, a safe standstill can also be determined. Longitudinal forces consist of quantities with known sign $F_{\mathrm{drive}}$ and quantities with velocity-dependent sign $F_{\mathrm{fric}}\left(v_{x}\right)$. The following torques and forces are taken into account on the vehicle:

$F_{\mathrm{engine}}$: engine force on the wheel $T_{\mathrm{engine}}\to F_{\mathrm{engine}}=T_{\mathrm{engine}}/r_{D}$;
$F_{\mathrm{brake}}$: brake force on the wheel $T_{\mathrm{brake}}\to F_{\mathrm{brake}}=T_{\mathrm{brake}}/r_{D}$;
$F_{\mathrm{drag}}$: engine drag force on the wheel $T_{\mathrm{drag}}\to F_{\mathrm{drag}}=T_{\mathrm{drag}}/r_{D}$;
$F_{\mathrm{roll}}$: rolling friction force $F_{\mathrm{roll}}=\mu_{\mathrm{roll}}\cdot m\cdot g\cdot \cos\left(\phi \right)$;
$F_{\mathrm{slope}}$: slope downforce $F_{\mathrm{slope}}=m\cdot g\cdot \sin\left(\phi \right)$.

The frictional forces act against the velocity and can be calculated by the sign function of the velocity, the braking force, the engine drag force, and the rolling friction force:

(10) $$\begin{array}{cc} F_{\mathrm{fric},\mathrm{abs}} & =F_{\mathrm{brake}}+F_{\mathrm{drag}}+F_{\mathrm{roll}},\\ F_{\mathrm{fric}}\left(v_{x}\right) & =\mathrm{sign}\left(v_{x}\right)\cdot F_{\mathrm{fric},\mathrm{abs}}. \end{array}$$

The driving force consists of the engine force and the inclination and depends on the direction of the velocity:

(11) $$F_{\mathrm{drive}}=F_{\mathrm{engine}}+F_{\mathrm{slope}}.$$

**Figure 4:**
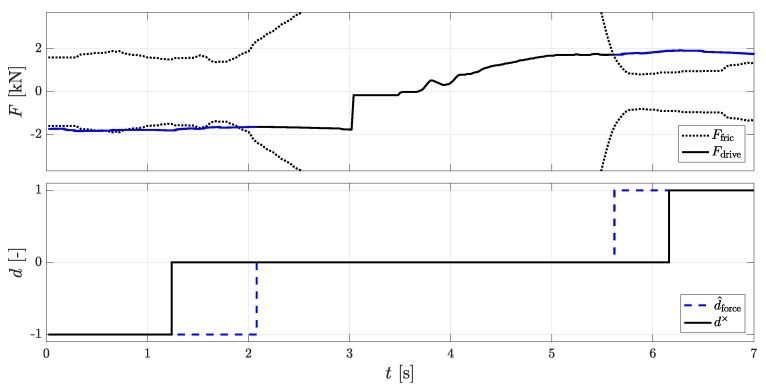
(**Top**): Calculated forces for the force equilibrium method for detection of rolling directions; (**bottom**): the rolling directions of the DWPC signal $d^{\times }$ and the force equilibrium method ${\hat{d}}_{\mathrm{force}}$.

In Figure 4 the driving force $F_{\mathrm{drive}}$ and the positive or negative value of frictional force $F_{\mathrm{fric}}$ from the force equilibrium are represented. As soon as the driving force $F_{\mathrm{drive}}$ exceeds the friction force $F_{\mathrm{fric}}$ a rolling direction is defined and the line in the top figure changes from blue to black. As soon as the driving force $F_{\mathrm{drive}}$ is smaller in amount than the frictional force $F_{\mathrm{fric}}$ and there is no DWPC for 200 ms a save standstill is defined.

This approach uses the slope that is not directly available on the vehicle bus, which is why it must be calculated. In the following, the method with which slope can be calculated is explained briefly.

### 3.5. Slope and Cross-Slope Calculation

The slope and cross-slope can be determined by an adjustment of the acceleration sensors with a calculated acceleration through the vehicle model [14]. The vehicle longitudinal acceleration $a_{x}$ can be determined by deriving the vehicle velocity:

(12) $$\begin{array}{cc} a_{x}^{*} & =\frac{dv_{x}}{dt}={\dot{v}}_{x}. \end{array}$$

The vehicle transverse acceleration $a_{y}$ can be determined by using the *Single-Track Model* from the tangential velocity $v_{x}$ and the instantaneous pole distance $r_{\mathrm{RM}}$ or the yaw rate ω:

(13) $$\begin{array}{cc} a_{y}^{*} & =\frac{v_{x}^{2}}{r_{\mathrm{RM}}}=\frac{v_{x}}{\omega }. \end{array}$$

When the vehicle is on a slope or cross-slope, the acceleration sensors measure vehicle acceleration relative to the road and induced downhill acceleration due to gravity g= 9.81 m/s${}^{2}$. It is assumed that the acceleration in the *z*-direction is $a_{z}^{*}=0$, since it is assumed that the vehicle does not drive in a loop or a steep curve when parking. With the help of the rotating matrix $T_{\phi ,\psi }$ the correlation results:

(14) $$\begin{array}{cc} a^{\times }=\left[\begin{array}{c} a_{x}^{\times }\\ a_{y}^{\times }\\ a_{z}^{\times } \end{array}\right] & =\left[\begin{array}{c} a_{x}^{*}\\ a_{y}^{*}\\ a_{z}^{*} \end{array}\right]+T_{\phi ,\psi }\cdot \left[\begin{array}{c} 0\\ 0\\ g \end{array}\right]=\left[\begin{array}{c} a_{x}^{*}\\ a_{y}^{*}\\ a_{z}^{*} \end{array}\right]+\underset{T_{\phi }}{\underset{︸}{\left[\begin{array}{ccc} 1 & 0 & 0\\ 0 & c\;\phi & s\;\phi \\ 0 & -s\;\phi & c\;\phi \end{array}\right]}}\cdot \underset{T_{\psi }}{\underset{︸}{\left[\begin{array}{ccc} c\;\psi & 0 & -s\;\psi \\ 0 & 1 & 0\\ s\;\psi & 0 & c\;\psi \end{array}\right]}}\cdot \left[\begin{array}{c} 0\\ 0\\ g \end{array}\right]\\ & =\left[\begin{array}{c} {\dot{v}}_{x}+g\cdot \sin\left(\phi \right)\\ \frac{v_{x}}{\omega }+g\cdot \sin\left(\psi \right)\cos\left(\phi \right)\\ g\cdot \cos(\psi )\cos(\phi ) \end{array}\right]. \end{array}$$

If the equation is solved, the tilt angle is ϕ and the tilt angle is ψ:

(15) $$\begin{array}{c} \phi =\arcsin\left(\frac{a_{x}-{\dot{v}}_{x}}{g}\right),\;\psi =\arcsin\left(\frac{a_{y}-\frac{v_{x}}{\omega }}{g\cdot \cos(\phi )}\right). \end{array}$$

With the conversion for the slope slope and cross-slope crossslope, one can obtain the following:

(16) $$\begin{array}{cc} slope & =\tan\left(\phi \right)\cdot 100,\\ crossslope & =\tan\left(\psi \right)\cdot 100. \end{array}$$

The slope slope is required for estimating the rolling direction. The cross-slope crossslope is no longer important in this work.

### 3.6. Result of Slope Calculation

**Figure 5:**
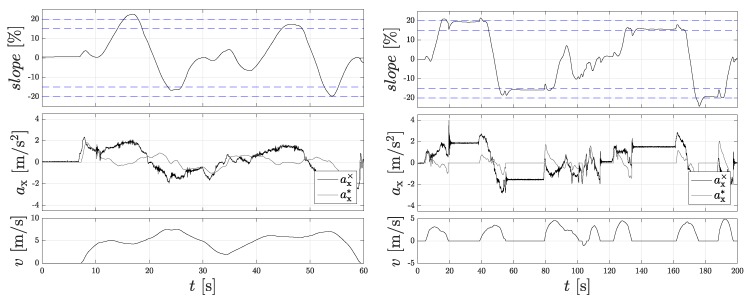
Slope estimation on the gradient hill. (**Left**): During the measuring run without standstill; (**Right**): with standstill. (**Top**): calculated slope slope; (**Middle**): measured acceleration of the IMU $a_{x}^{*}$ and acceleration of the filtered signal of the wheel speed sensors $a_{x}^{*}$; (**Bottom**): vehicle velocity *v*.

To validate the slope calculation, test drives were performed on a test hill with a defined gradients of 15% and 20%. In front of and behind the hill, the ground is flat. It is assumed that the zero-point error has already been corrected by a sensor calibration at the start of the vehicle in the flat plain. Figure 5 (left) shows the results for the first measurement on the hill. Here, the gradient hill was crossed faster and not stopped within the gradient. The dashed lines indicate the areas with 15% and 20% gradients, respectively.

Due to the faster gradient changes, the actual gradient is only reached very briefly or not completely. A glance at the individual accelerations shows that the filtered signal of the wheel speed sensors $a_{x}^{*}$ can hardly follow the measured acceleration signal. In the 2nd measurement, the gradient hill was slowly crossed over and stopped within the gradient. Figure 5 (right) shows the measured slope slope. It is conspicuous that the signal swings out before reaching the provided gradient values, which is due to the fact that the longitudinal acceleration calculated by the wheel speed sensors is already fixed to 0, but the acceleration sensor still measures the pitching of the car body. These vibrations have subsided after 3 s at the latest, but cannot be avoided due to the position of the IMU in the vehicle. Further sources of error are a permanent pitching of the vehicle on the runway, which leads to an unintentional alignment of the sensor and inaccurate sensor correction values. Sensor errors can cause a zero-drift error at an angle of ϕ = 11.31 ${}^{\circ }$ and sensitivity errors of Δϕ = 4.09${}^{\circ }$ and Δϕ = 0.34${}^{\circ }$. The linearity and hysteresis errors are not taken into account due to the small deflections and the lack of dynamics when starting from standstill for the detection of the rolling direction due to the force equilibrium in the longitudinal direction of the vehicle. New inertial sensors usually provide a value for the zero-point drift correction, which is required for an accurate slope estimation.

## 4. Initialization Logic of the Dual-Localization Filter

When the AMR+ rolling direction indicates a driving direction, the primary (1st) localization filter follows the estimated direction and the secondary (2nd) localization filter follows the opposite direction. If the AMR+ rolling direction is defined, both localization filters follow the same direction. The system determines which localization filter has previously moved in the correct direction and describes the initialization state in more detail at all times. In total, seven different initialization states (Init) can be assumed (see Table 1).

1. Initial state: no DWPC and no estimated direction.
2. Active state: there is no DWPC yet, but there is already an estimated direction. Both localization filters move in opposite directions.
3. Passive state: no DWPC and no estimated direction. Before, however, there was an initial direction.
4. Ideal state: there is DWPC and the AMR+ roll direction immediately recognizes a rolling direction.
5. Secondary initialization state: there is DWPC and the estimated direction was correct. The 2nd localization filter is initialized with the values of the 1st localization filter.
6. Primary initialization state: there is DWPC and the estimated direction was wrong. The 1st localization filter is initialized with the values of the 2nd localization filter.
7. Error state: DWPC appears and the AMR+ roll direction immediately changes the sign.

**Figure 6:**
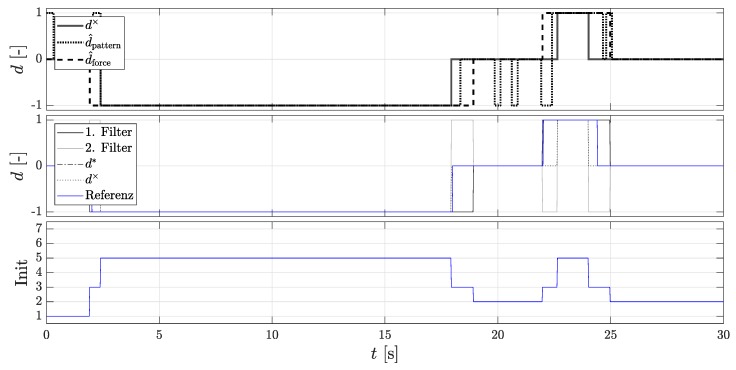
Procedure of the Dual-Localization filter for a complete parking process. (**Top**): The rolling directions of the DWPC signal $d^{\times }$, the pattern recognition method ${\hat{d}}_{\mathrm{pattern}}$, and the force equilibrium method ${\hat{d}}_{\mathrm{force}}$; (**Middle**): rolling directions of the 1st and the 2nd localization filter, the fused estimated rolling direction of the new methods $d^{*}$, the rolling direction of the DWPC signal and the reference direction; (**Bottom**): Initialization states.

Figure 6 shows the procedure of the Dual-Localization filter and the change of the initialization states. The figure above shows the rolling direction signals of the Dual-Localization filter for a complete parking process consisting of a parking in and parking out process. The middle figure shows the directional estimation. The figure below shows the time course of the initialization state signal from Table 1.

In the first seconds, the vehicle is stationary and all rolling direction signals are 0 or point in the initial forward direction, the initial state (Init 1) prevails. From 2 s, a backward estimation direction is estimated and the AMR+ rolling direction is still 0. The active state (Init 2) prevails, the 1st localization filter takes the estimated direction, and the 2nd localization filter the opposite direction. From 3 s, an AMR+ rolling direction is detected, so the 2nd initialization state (Init 5) is selected and the 2nd localization filter is initialized with the values of the 1st localization filter. From 18 s, the AMR+ rolling direction jumps to 0, and the direction estimation returns the value −1, so the active state (Init 2) is activated again and the 2nd localization filter takes the opposite direction of the 1st localization filter. From 19 s, the direction estimate returns the value 0 and the vehicle is at a standstill. Since the AMR+ rolling direction and the direction estimation are set to 0, it is not possible to evaluate which localization filter has moved in the true direction because the passive state (Init 3) is present. After that, the pattern for the forward movement is exactly the same.

## 5. Results

### 5.1. Assessment of the Concept during Parking

**Figure 7:**
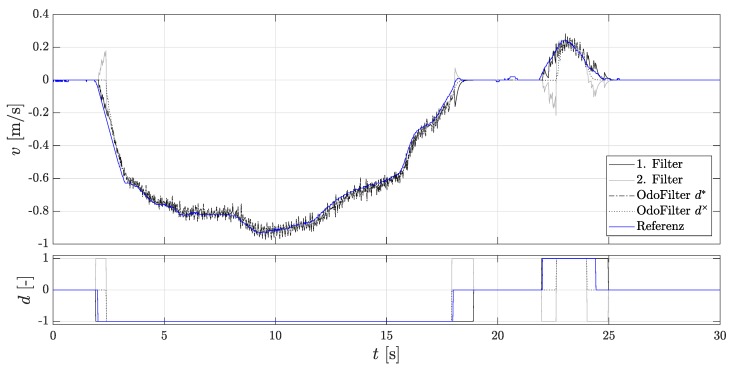
Velocities of the Dual-Localization filter during parking. (**Top**): The velocity of the 1st and the 2nd localization filter, the velocity of the localization filter using the fused estimated rolling direction of the new methods, the velocity of the localization filter using the rolling direction of the DWPC signal, and the reference velocity; (**Bottom**): Rolling directions.

To test the performance of the Dual-Localization filter, a localization filter that always uses the estimated direction and one that uses the AMR+ rolling direction are used. Figure 7 shows the time line of the velocity.

**Figure 8:**
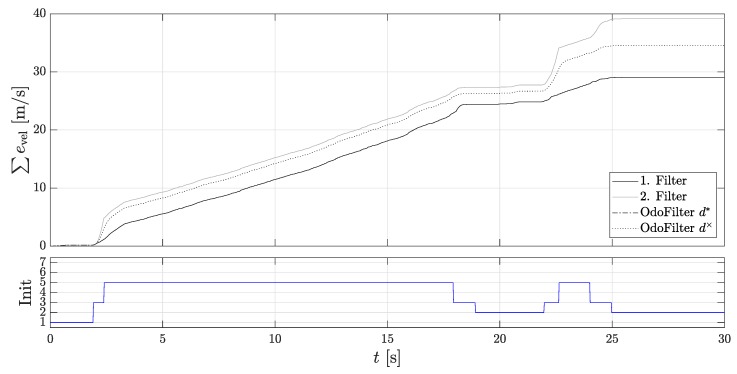
Accumulated velocity errors of the Dual-Localization filter during parking. (**Top**): The accumulated velocity errors of the 1st and the 2nd localization filter, of the localization filter using the fused estimated velocity of the new methods, and of the velocity of the localization filter using the rolling direction of the DWPC signal; (**Bottom**): Initialization states.

It can be seen that the 2nd localization filter is initialized after each start-up because the estimated direction was correct. Here, one sees the performance of the methods presented in Section 3 for faster detection of rolling direction. In addition, the accumulated velocity error is considered for the evaluation, since the assessment criteria of comparison of the position gives an incorrect impression, as they do not explicitly consider the rolling direction. An incorrectly assumed rolling direction signal can compensate for the position error. Figure 8 shows the accumulated velocity error. Here, the 1st localization filter and the comparison filter with the estimated direction achieve the same very good performance.

The 2nd localization filter shows the largest accumulated velocity error. However, this is always initialized with the states of the 1st localization filter, so both filters have the same initialization state values when the AMR+ rolling direction is present. The accumulated velocity error built up previously with an unavailable AMR+ rolling direction cannot be reduced. The accumulated error of the 1st localization filter is lower compared to the localization filter with the AMR+ rolling direction. This confirms that the directional estimation, as far as it corresponds to the true direction, makes sense and leads to a more accurate modeling of the localization filter.

### 5.2. Assessment of the Concept on the Hill

**Figure 9:**
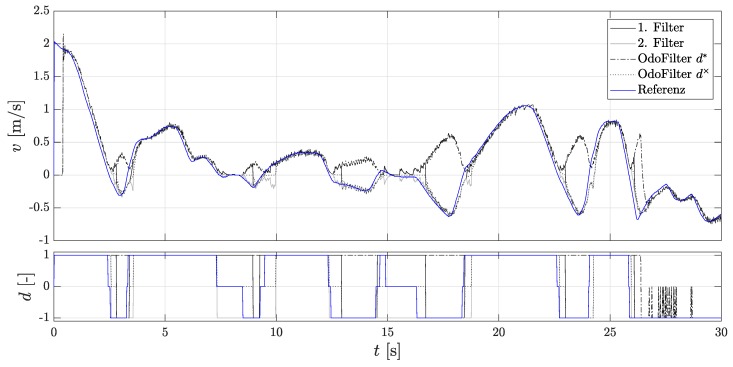
Velocities of the Dual-Localization filter on the slope hill. (**Top**): The velocity of the 1st and the 2nd localization filter, the velocity of the localization filter using the fused estimated rolling direction of the new methods, the velocity of the localization filter using the rolling direction of the DWPC signal, and the reference velocity; (**Bottom**): Rolling directions.

As described in Section 3.5, problems with the estimated slope can occur especially on a hill if the acceleration sensor is not sufficiently calibrated. In order to ensure that both approaches reach their limits for estimating the rolling direction, multiple starts are made on the incline or fast rolling direction changes are induced. In Figure 9 the velocity progression and the rolling directions are shown again.

**Figure 10:**
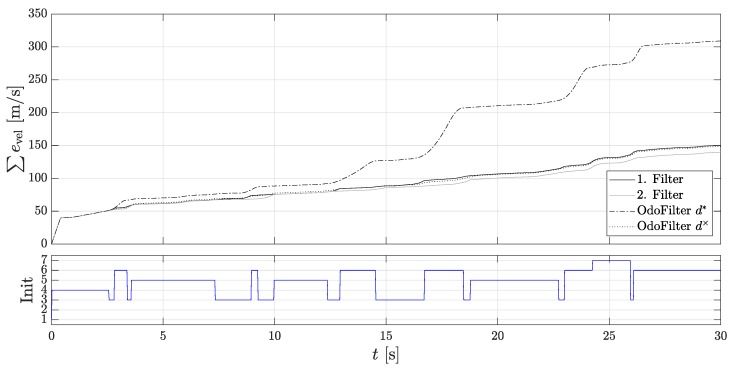
Accumulated velocity errors of the Dual-Localization filter on the slope hill. (**Top**): The accumulated velocity errors of the 1st and the 2nd localization filter, of the localization filter using the fused estimated velocity of the new methods, and of the velocity of the localization filter using the rolling direction of the DWPC signal; (**Bottom**): Initialization states.

It can be seen that the 2nd localization filter usually uses the right direction here, since the estimated rolling direction is wrong. Especially the approach with the force equilibrium seems to be erroneous here, because in some cases it is approached backwards and the incline is difficult to determine. Figure 10 shows again the accumulated velocity error. This is as expected the smallest for the 2nd localization error, since it always uses the opposite rolling direction.

**Figure 11:**
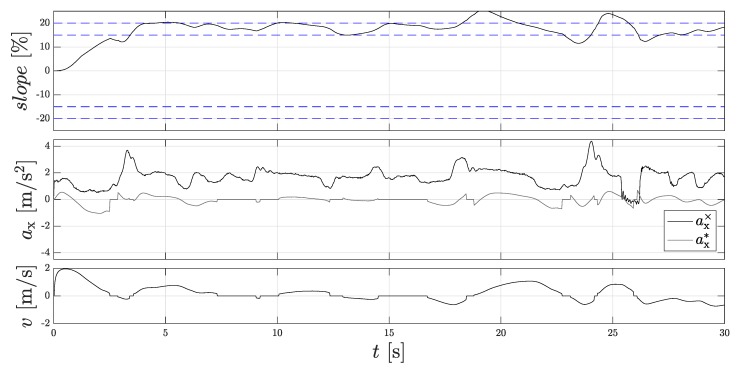
Slope estimation on the gradient hill during the measuring run with special driving style. (**Top**): Calculated slope slope; (**Middle**): Measured acceleration of the IMU $a_{x}^{*}$ and acceleration of the filtered signal of the wheel speed sensors $a_{x}^{*}$; (**Bottom**): Vehicle velocity *v*.

The investigations have shown that the rolling direction recognition in the flat plane estimates the direction well, but if there is a slope, the estimated direction might be wrong. Therefore, the approach to the force equilibrium is strongly dependent on the gradient. Figure 11 shows the slope estimation.

At the beginning of the measurement, the vehicle stands still. Due to the reference measurement system, a negative zero offset of −1.3${}^{\circ }$ can be detected, since an acceleration is measured at standstill, which one can read at 0 s. In the first 3 s, the vehicle drives up the 20% gradient hill at about 2 m/s, which can be seen from the increasing angle of inclination and the increasing vehicle speed. From 5 s upwards, the maximum gradient angle of 20% is reached (see reference curve). The vehicle is then braked to a standstill. As the process continues, the vehicle is continuously started and braked. In addition, the vehicle can also roll back easily from time to time, which can be deduced from the negative velocities. The maximum value here is 28%, and the minimum value is 12%. Since the estimated slope changes with permanent starting and braking, this also has an influence on the estimated direction. The estimated direction is determined by the sign of the resulting force equilibrium. As a result, the constantly changing force direction can lead to an error in the estimated direction. The Dual-Localization filter structure counteracts this, since the evaluation logic detects the wrong estimated direction as soon as an AMR+ rolling direction is present again.

## 6. Conclusions

In this paper, an extension for a Bayesian localization filter, which calculates a lower speed error with unclear direction signals, is presented. New methods have been introduced to detect the rolling direction more quickly. To manage the localization filter in real time without a signal, a dual localization filter was additionally developed, which operates the localization filter in real time if direction signals are wrongly estimated. The results show the special feature on the reference gradient hill, and in the case of increased requirements, the double filter approach brings advantages in reducing the velocity error.

## Figures and Tables

**Table 1:** Initialization states of the initialization logic of the Dual-Localization filter.

Init	DWPC	$d^{\times }$	$d^{*}$	True/False
1	0	-	-	-
2	0	0	1	-
3	0	0	0	-
4	1	1	-	-
5	1	1	1	true
6	1	1	−1	false
7	1	−1	0	false
